# P-1015. Inpatient outcomes of Clostridioides Difficile infections in patients with Left Ventricular Assist Devices: A National Database Analysis

**DOI:** 10.1093/ofid/ofaf695.1211

**Published:** 2026-01-11

**Authors:** Muskaan Abdul Qadir, Hafsa Khan Tareen, Sarim Raheel, Zulfiqar H Jogezai, Ayesha Rashid, Bilal Siddiqui, Armaghan-e-Rehman Mansoor

**Affiliations:** University of New Mexico, Albuquerque, NewMexico; Aga Khan University, Multan, Punjab, Pakistan; Aga Khan University, Multan, Punjab, Pakistan; The Aga Khan University, New York City, New York; Allina Health, University of Minnesota, St. Paul, Minnesota; Midwestern University, Naperville, Illinois; University of Kentucky, Lexington, KY

## Abstract

**Background:**

Left ventricular assist devices (LVAD) carry a risk of device component infection, and deep-seated or recurrent infections often require long-term antibiotics. Antibiotic use is associated with *Clostridioides difficile* infection (CDI), which can cause substantial morbidity and result in inpatient stays. This study aims to evaluate the incidence, epidemiology and outcomes of inpatient admissions for CDI in patients with LVADs.

**Table 1:**
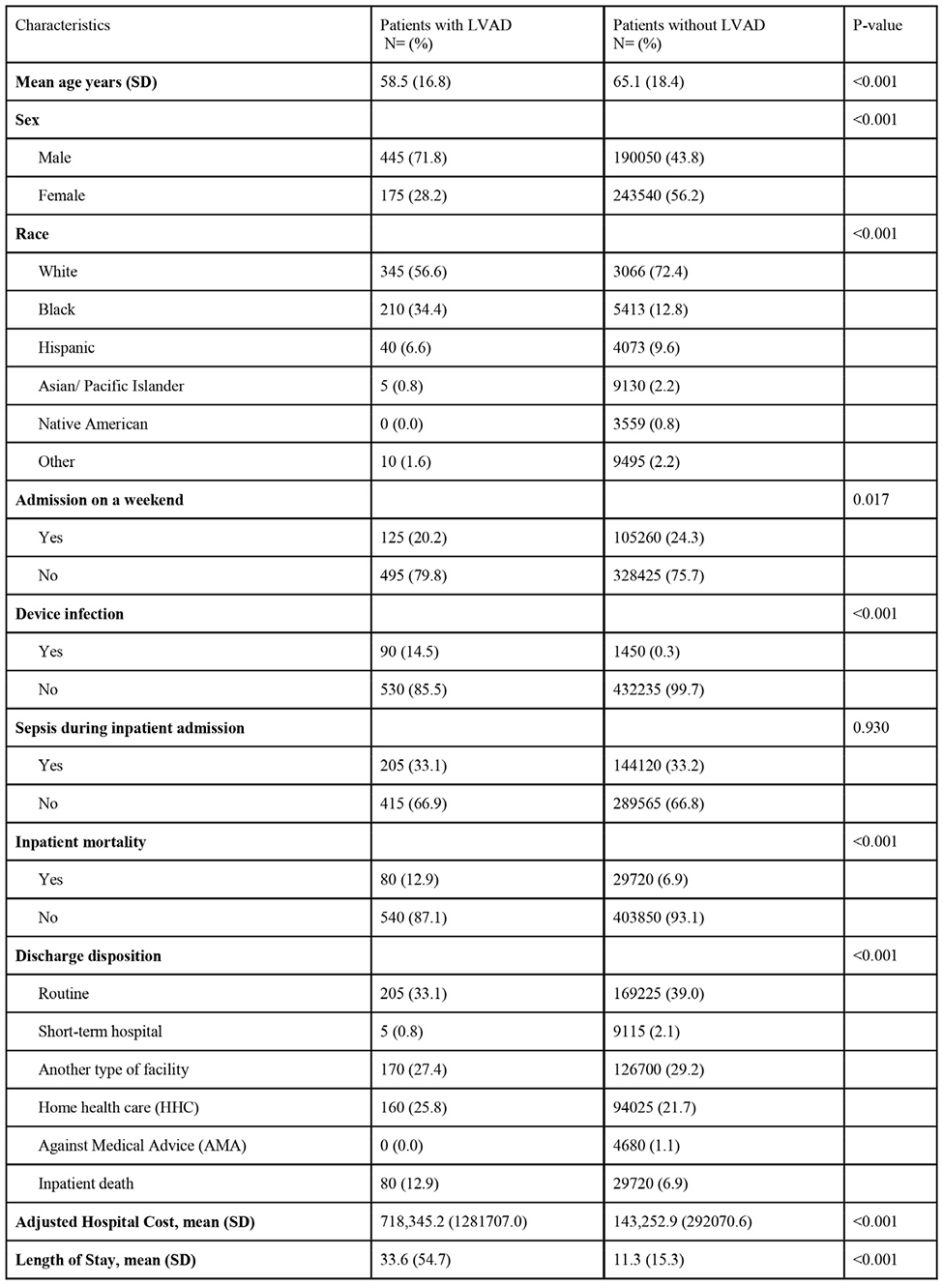
Demographics and Characteristics of patients with C. difficile infection; LVAD: left ventricular assist device

**Methods:**

The National Inpatient Sample (NIS) was queried for all inpatient admissions with a primary or secondary diagnosis of CDI between January 2021 to December 2022. Patients with an LVAD were identified using ICD-10 procedure and diagnosis codes. Demographics, outcomes, length of stay, and inpatient costs were recorded and analyzed using SPSS (v. 30, IBM). Weights assigned in NIS were used to calculate national estimates. Demographics and outcomes for patients with and without LVAD were compared using chi-square, and logistic regression performed to determine variables associated with inpatient mortality in the LVAD cohort.

**Results:**

A total of 432,235 admissions with a primary or secondary diagnosis of CDI were reported, of whom 530 (0.1%) occurred in patients with LVAD (Table 1). Patients with LVAD and CDI were younger (mean 58.5 vs. 65.1 years), more likely to be male (71.8% vs. 43.8%, p >0.001) and more likely to have a device-related infection (14.5% vs 0.3%, p< 0.001). Patients with LVAD had significantly higher in-hospital mortality (12.9% vs. 6.9%, p< 0.001), longer hospital stays (mean 54.7 vs. 15.3 days, p< 0.001), and higher cost of inpatient stay (mean $718,345 vs. $143,253, p< 0.001). In the LVAD cohort, females had lower inpatient mortality (OR 0.55, CI 0.30-0.99, p=0.044).

**Conclusion:**

Inpatient admissions for CDI in patients with LVAD were a small proportion of all CDI episodes, however these patients had significantly higher inpatient mortality, length of stay and inpatient cost compared to patients without LVAD. Given the frequent need for prolonged antibiotic use in patients with LVADs, clinicians should remain vigilant about CDI risk and its potentially severe outcomes in this vulnerable population.

**Disclosures:**

All Authors: No reported disclosures

